# COMPLICATIONS AFTER TRANSABDOMINAL SOAVE’S PROCEDURE IN CHILDREN WITH HIRSCHSPRUNG’S DISEASE

**DOI:** 10.1590/0102-672020180001e1421

**Published:** 2019-02-07

**Authors:** Shahnam ASKARPOUR, Mehran PEYVASTEH, Mohammad Hossein IMANIPOUR, Hazhir JAVAHERIZADEH, Saeed HESAM

**Affiliations:** 1 Department of Pediatric Surgery,; 2 Alimentary Tract Research Center; 3 Department of Biostatistics, Ahvaz Jundishapur University of Medical Sciences, Ahvaz, Khouzestan, Iran

**Keywords:** Constipatio, Hirschsprung diseas, Enterocoliti, Constipação intestina, Enterocolit, Doença de Hirschsprung

## Abstract

**Background:**

Hirschsprung’s disease is a congenital disorder that causes functional obstruction of large bowel.

**Aim:**

To evaluate complication and bowel function score of children with Hirschsprung’s disease who underwent transabdominal Soave’s procedure.

**Methods:**

In this study all the children with Hirschsprung’s disease who underwent transabdominal Soave procedure were evaluated regarding bowel function and complication of trans-abdominal Soave’s procedure.

**Results:**

Were enrolled 160 children. Enterocolitis and constipation were seen in 15% of the cases. Fecal incontinency was the least frequent study which was seen in 1% of the children.

**Conclusion:**

Constipation and enterocolitis was the most frequent complication following transabdominal Soave technique**.**

## RESUMO

**Racional:**

A doença de Hirschsprung é um distúrbio congênito que causa obstrução funcional do intestino grosso.

**Objetivo:**

Avaliar as complicações e o escore de função intestinal de crianças com a doença submetidas ao procedimento transabdominal de Soave.

**Métodos:**

Neste estudo, todas as crianças com doença de Hirschsprung submetidas ao procedimento transabdominal de Soave foram avaliadas quanto à função intestinal e complicação do procedimento.

**Resultados:**

Foram incluídas 160 crianças. Enterocolite e constipação foram observadas em 15% dos casos. A incontinência fecal foi menos frequente e observada em 1% das crianças.

**Conclusão:**

Obstipação e enterocolite foram as complicações mais frequentes após a técnica de Soave transabdominal em crianças.

## INTRODUCTION

Hirschsprung’s disease is a congenital disorder that causes functional obstruction of large bowel. It´s incidence is estimated in 1:5000 live birth with a male predominance^1^ ^,^ ^2^. Diagnosis is done using anorectal manometry, barium enema^3^ and rectal biopsy. Niramis et al^12^ with patients who underwent pull-through procedure, found enterocolitis as the most common post-surgical complication. For Little et al.^9^ enterocolitis was the most common post-operative complication followed by constipation and bowel obstruction. In the study by Shakya et al^16^, constipation was seen in 11.7% of children who underwent transabdominal Soave’s pull-through procedure. In the literature review by Rintala et al^15^, fecal incontinency and constipation were the most post-operative complication of Hirschsprung’s disease. Bowel function was lower than normal population. 

The aim of this study was to evaluate complications and bowel function score in children with Hirschsprung’s disease who underwent transabdominal Soave’s procedure.

## METHODS

This study was approved by Ethical Committee of the University (IRAJUMS.REC.1395.364). It was approved by Research Affair of Ahvaz Jundishapur University of Medical Sciences.

All children who underwent trans-abdominal Soave’s procedure were included. Patients with Down syndrome and total colonic involvement were excluded. 

**FIGURE 1:**
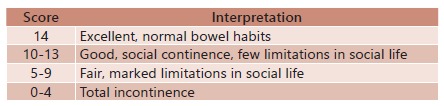
Functional score for clinical evaluation

Qualitative clinical scoring was used for assessment of bowel function which was proposed by Holschneider^4^. There is no need for physical examination. According to these criteria, 14 points means excellent bowel function Score interpretation is shown in Figure 1.

## RESULTS

In this study, 160 children with Hirschsprung disease who underwent Soave’s procedure were included. Results of postoperative complications are in Table 1 and show constipation (n=24,15%) and enterocolitis (n=24, 15%) being the more frequent complications after trans-abdominal Soave’s procedure. The least one was fecal incontinency which was seen in 1% (n=2) of the cases. The patients´ score is seen in Table 3.

**TABLE 1:** Complications following transabdominal Soave’s procedure

Complication	n (%)
Enterocolitis	24 (15%)
Fecal incontinency	2 (1%)
Constipation	24 (15%)
Anastomotic stricture	11 (7%)
Anastomotic leak	6 (4%)

Initially were included 163 children who underwent surgery during two years. Among them, two who had Down syndrome and one total aganglionosis were excluded. So, the total enrollment was 160 children. There were 108 (67.5%) male and 52 (32.5%) female. Most of them (n=96, 60%) were diagnosed when they had less than one month of age (Table 2). As seen in Table 2, most of the patients underwent procedure at the ages <1 month. 

**TABLE 2:** Age distribution at diagnosis

Age	n (%)
<1month	96 (60%)
1-6 month	37 (23%)
6-12month	19 (11%)
12 month-5 years	8 (5%)

**TABLE 3:** Evaluation of patients according to bowel function score

Score	n (%)
14	123 (77%)
10-13	24 (15%)
5-9	11 (7%)
0-4	2*(1%)

## DISCUSSION

Most of our cases were diagnosed and underwent surgery in the neonatal period, contrary to the results published by Mabula et al^10^ referring only 5.5% in this condition. In the developed countries, more than 90% of the cases were in the neonatal period. So, our findings are consistent with developed countries, as discussed by Archibong^2^.

In this study 67.5% of the patients were boys and 32.5% girls. De Lor gin et al^3^ and Martucciello^11^, the number of boys/girls were reported about 4/1 which, so higher than here.

Enterocolitis and constipation were the most common postoperative complication as also referred by other authors^7^ ^,^ ^10^. The rate of enterocolitis in this study was higher than the one reported by Parahita et al.^13^ and Huang et al also mentioned enterocolitis as the earliest postoperative complication (28.73%) being fecal incontinency (20.99%) also frequent^5^. Constipation can be caused by high anal resting pressure and a weak rectal peristalsis as described by Keshtgar et al^6^.

Fecal incontinency was reported in 1% of our cases, differently to the ones reported by Niramis et al^12^ in 15.6% with the procedure^4^. Possibly, poor surgical technique could be the contributing factor for fecal incontinency ^7^. 

Constipation was seen in 15% of children in this study. It has different results in the literature, as Niramis et al^12^ that reported it´s presence in 8.5%, lower than in our study. 

The same divergence can be seen with the anastomotic stricture. In our sample it occurred in 7% different from Niramis et al data with 17.1%^12^. 

The main limitations of this study were being in a single center and relatively in short follow-up. Another multicenter study with longer follow-up is recommended to obtain more reliable results.

## CONCLUSION

Constipation and fecal incontinency were the most frequent complication following transabdominal Soave’s procedure in follow-up of two years 
